# Tranexamic acid in traumatic brain injury: an explanatory study nested within the CRASH-3 trial

**DOI:** 10.1007/s00068-020-01316-1

**Published:** 2020-02-19

**Authors:** The CRASH-3 Intracranial Bleeding Mechanistic Study Collaborators, Abda Mahmood, Kelly Needham, Haleema Shakur-Still, David Davies, Antonio Belli, Sabariah Faizah Jamaluddin, Tim Harris, Fatahul Laham Mohamed, Caroline Leech, Hamzah Lotfi, Phil Moss, Phillip Hopkins, Darin Wong, Jason Kendall, Adrian Boyle, Mark Wilson, Melanie Darwent, Ian Roberts

**Affiliations:** Clinical Trials Unit, Faculty of Epidemiology and Population Health, London School of Hygiene and Tropical Medicine, Keppel Street, London, WC1E 7HT UK

**Keywords:** Traumatic brain injury, Tranexamic acid

## Abstract

**Purpose:**

The CRASH-3 trial is a randomised trial of tranexamic acid (TXA) on death and disability in patients with traumatic brain injury (TBI). It is based on the hypothesis that early TXA treatment can prevent deaths from post-traumatic intracranial bleeding. The results showed that timely TXA treatment reduces head injury deaths in patients with reactive pupils and those with a mild to moderate GCS at baseline. We examined routinely collected CT scans in a sample of 1767 CRASH-3 trial patients to explore if, why, and how patients are affected by TXA.

**Methods:**

The CRASH-3 IBMS is an explanatory study nested within the CRASH-3 trial. We measured the volume of intracranial bleeding on CT scans using established methods (e.g. ABC/2).

**Results:**

Patients with any un-reactive pupil had a median intracranial bleeding volume of 60 ml (IQR 18–101 ml) and patients with reactive pupils had a median volume of 26 ml (IQR 1–55 ml). Patients with severe GCS had median intracranial bleeding volume of 37 ml (IQR 3–75 ml) and patients with moderate to mild GCS had a median volume of 26 ml (IQR 0.4–50 ml). For every hour increase from injury to the baseline scan, the risk of new bleeding on a further scan decreased by 12% (adjusted RR = 0.88 [95% CI 0.80–0.96], *p* = 0.0047).

**Conclusion:**

Patients with reactive pupils and/or mild to moderate GCS may have benefited from TXA in the CRASH-3 trial because they had less intracranial bleeding at baseline. However, because bleeding occurs soon after injury, treatment delay reduces the benefit of TXA.

## Introduction

The CRASH-3 trial is a multi-centre, randomised, placebo-controlled trial of the effects of tranexamic acid on death and disability in patients with traumatic brain injury (TBI) [1]. Adults with TBI who were within 3 h of their injury and had a Glasgow coma scale score (GCS) ≤ 12 or any intracranial bleeding on CT scan were included in the primary analysis. We hypothesised that early administration of tranexamic acid might prevent deaths from post-traumatic intracranial bleeding. We found that rapid tranexamic acid treatment reduces head injury deaths in patients with mild to moderate head injury (RR = 0.78 95% CI 0.64–0.95) but there was no apparent reduction in severe head injury (RR = 0.99, 95% CI 0.91–1.07), regardless of time to treatment. Because our main aim was to assess the effect of tranexamic acid on head injury death, to simplify the trial procedures, we did not plan to collect data on the amount of intracranial bleeding in all patients. However, while the trial was underway, the data monitoring committee asked us to consider collecting these data on a sample of trial patients “to explore if, why, and how patients are affected by tranexamic acid.” In response, routinely collected brain imaging data (mainly CT scans) were assessed in 1767 CRASH-3 trial patients. These patients were scanned before and/or after randomisation. Because early TXA treatment is expected to be more effective than late treatment [2], to reduce time to randomisation, many patients were randomised into the CRASH-3 trial before CT (i.e. not all 1767 patients in the IBMS had their hospital admission scan done before randomisation). A total of 1147 patients in the IBMS had a baseline (prior to randomisation) CT scan, of whom 812 patients had another clinically indicated brain scan. We measured the volume of intracranial bleeding on all scans using established methods (e.g. ABC/2) [3] and collected data on other CT features of TBI. Here we consider the CRASH-3 trial results in light of the CT scan data.

The main aim of this paper is to describe the occurrence of intracranial pathologies (especially intracranial bleeding) at baseline. In patients who are re-scanned after randomisation into the trial, the data has been collapsed across treatment groups. This is because there are important methodological flaws when using routinely collected scans to explore the effect of tranexamic acid on intracranial bleeding and other endpoints measured on post-randomisation imaging. Scans may have been done after randomisation because a patient did or did not receive tranexamic acid, and so treatment effect estimates are at high risk of bias. This critique is beyond the scope of the current paper and so will not be presented here.

## Methods

Detailed protocols for the CRASH-3 trial and Intracranial Bleeding Mechanistic Study (IBMS) are published separately [4, 5]. These will be briefly summarised here.

Eligible patients were randomly allocated to receive tranexamic acid or matching placebo (0.9% sodium chloride) by intravenous infusion. Baseline information was collected on the trial entry form. This included an assessment of injury severity using the GCS (eye, verbal and motor responses) and pupil reaction (both react, one reacts, none react). After the trial entry form was complete, the lowest-numbered treatment pack remaining from a box of eight treatment packs was taken. If the ampoules inside the treatment pack were intact, the patient was considered randomised into the trial. Entry form data were entered into a secure online database by the trial investigators. Patients and study staff (site investigators and trial coordinating centre staff) were masked to allocation. Once randomised, outcome data were collected even if the treatment was not given (in accord with the intention to treat principle). Outcome data were collected 28 days after randomisation, at discharge from the randomising hospital, or at death (whichever was first).

The CRASH-3 IBMS is an explanatory study nested within the CRASH-3 trial. Patients who fulfilled the eligibility criteria for the CRASH-3 trial, with a GCS of 12 or less or intracranial bleeding on a CT scan done before randomisation, were eligible for inclusion in the IBMS. Routinely collected CT scans were manually examined on hospital software (Picture Archiving and Communication System) between February 2016 and January 2019 across 14 hospitals in the UK and Malaysia. Most patients in the IBMS were randomised into the CRASH-3 trial within 3 h of injury (76%, *n* = 1350); the rest were randomised between 3 and 8 h of injury. Patients had a median age of 45 years (IQR 29–63), median systolic blood pressure of 136 mmHg (IQR 120–155), and median GCS of 7 (IQR 3–10) (80% male, 20% female). In the CRASH-3 IBMS, a total of 65% of patients (*n* = 1147) had a baseline CT scan done within a median of 2 h after injury (IQR 1–2 h), of whom 71% had another clinically indicated brain scan done within a median of 35 h after injury (IQR 19–77 h).

Simple validated scales were used to estimate intracranial haemorrhage volume on CT scans. The ABC/2 method is a quick and easy technique used to estimate intracranial haemorrhage volume. This method selects a representative slice near the centre of the haematoma on which the bleed is most visible. On this slice, two measurements are taken: (A) the maximal diameter; (B) width perpendicular to A. For the measurement of depth, the maximal number of slices on which the haematoma is visible is multiplied by slice thickness (C). These three measurements are multiplied and the sum divided by two (ABC/2) to provide the volume measurement in cm^3^ (ml). One cubic centimetre is equivalent to one millilitre. Intra-parenchymal bleeding, epidural bleeding and intra-ventricular bleeding volumes were estimated using the ABC/2 method [5]. Because the ABC/2 method assumes haemorrhage has an almost spherical shape, an alternative method was used to estimate subdural bleeding volume, which is typically crescent shaped. Subdural bleeding volume was estimated using its maximum diameter [5]. The occurrence of mass effect (sulcal effacement, ventricular effacement, midline shift) was also examined using scans and their accompanying radiology reports, which were rated at each hospital site by one outcome assessor. Anonymised scan ratings were entered into a web database developed for the purpose of the IBMS. All data were cleaned prior to analysis in statistical package Stata.

## Results

### Intracranial bleeding on baseline CT scan

**Fig. 1:**
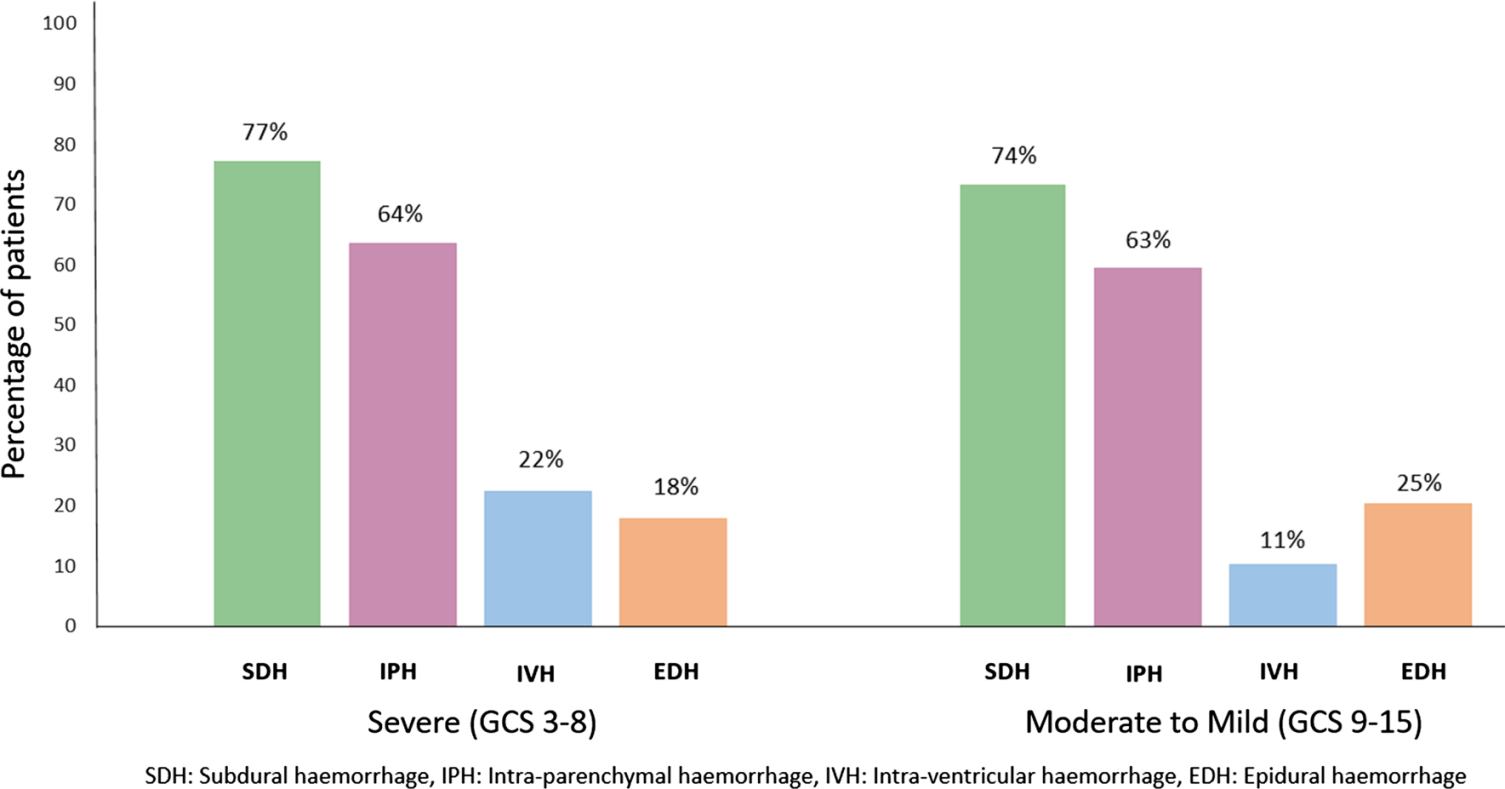
Baseline prevalence and type of intracranial bleeding by Glasgow Coma Score (GCS)

Figure 1 shows the type and frequency of intracranial bleeding on baseline CT scans according to baseline GCS. A total of 61% of patients with a baseline scan presented with more than one type of bleed. With the exception of epidural bleeding, which was more prevalent in patients with mild to moderate GCS, all other bleed types were more common in patients with a severe GCS. Subdural bleeds had a larger median volume of 46 ml (IQR 27–71 ml) compared to epidural bleeds with 6 ml (IQR 2–20 ml), intra-parenchymal bleeds with 1 ml (IQR 0.2–3 ml), and intra-ventricular bleeds with a median volume of 0·4 ml (IQR 0.1–2 ml).

**Fig. 2:**
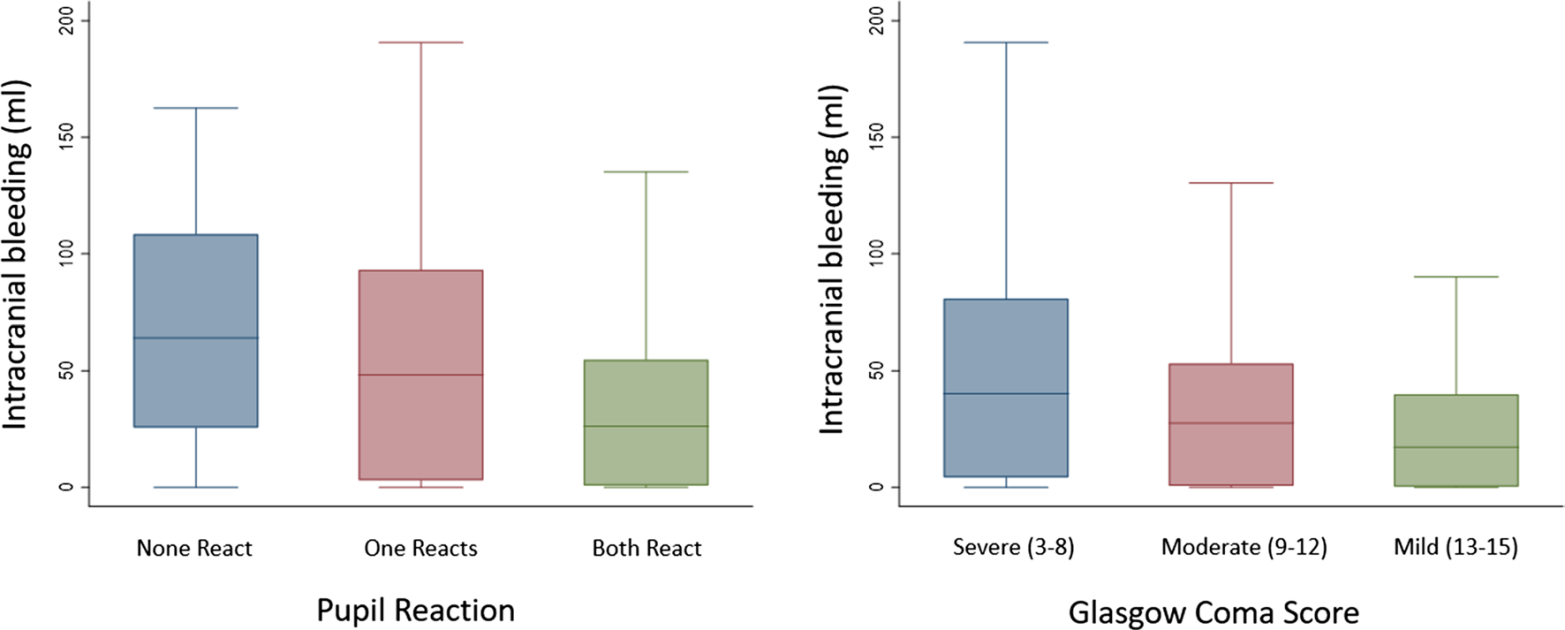
Baseline intracranial bleeding volume distribution

Figure 2 shows the volume distribution of intracranial bleeding on baseline CT scans by pupil reactions and GCS. The median volumes of 64 ml (IQR 26–108 ml) in patients with no reactive pupils and 48 ml (IQR 3–93 ml) in patients with one reactive pupil were larger than 26 ml (IQR 1–55 ml) in patients with two reactive pupils. The median volumes of 37 ml (IQR 3–75 ml) in patients with a severe GCS were greater than 28 ml (IQR 1–53 ml) for moderate GCS and 18 ml (IQR 0.2–41 ml) in mild GCS. But there is substantial overlap in bleeding volumes between pupil reaction groups and GCS groups.

We used data on the time of injury and time of CT scan to estimate the time-adjusted volume of intracranial bleeding. Table 1 shows the time-adjusted volume of bleeding by pupil reaction, GCS score, and type of bleed. The time-adjusted volume of bleeding was largest in those with un-reactive pupils and in those with severe GCS. Subdural bleeding was more rapid than epidural, intra-parenchymal, and intra-ventricular bleeding.

**Table 1:** Baseline intracranial bleeding volume (adjusted for time from injury to baseline scan)

	Median (lower quartile, upper quartile) millilitres/hour
All patients (*n* = 1135)	16 (1, 36)
**Pupil reaction**	
None react (*n* = 141)	32 (14, 55)
One react (*n* = 94)	21 (2, 47)
Both react (*n* = 867)	13 (0.5, 31)
**Glasgow coma scale (GCS) score**^**a**^	
Severe (*n* = 388)	20 (2, 41)
Moderate (*n* = 331)	13 (0.3, 29)
Mild (*n* = 91)	8 (0.1, 20)
Bilateral un-reactive pupils or GCS 3^a^ (*n* = 131)	28 (10, 54)
**Type of intracranial bleeding**	
Subdural (*n* = 732)	25 (13, 42)
Epidural (*n* = 215)	4 (1, 10)
Intra-parenchymal (*n* = 709)	0.4 (0.1, 2)
Intra-ventricular (*n* = 184)	0.3 (0.1, 1)

^a^Glasgow Coma Scale (GCS) score assessed before intubation / sedation (*n* = 814/1135) (72%)

**Fig. 3:**
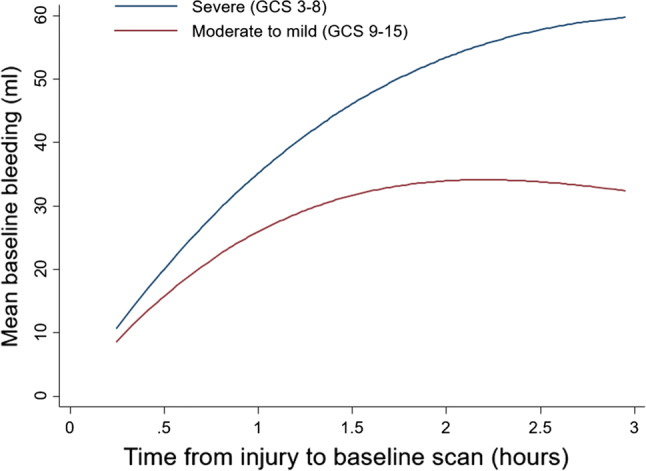
Association between time from injury to baseline scan and intracranial bleeding on baseline scan

But the bleeding rate may not be constant. We found a non-linear association between time and bleeding volume (see Fig. 3). The majority of expansion occurred in the first 1–1.5 h after injury. Patients with a severe GCS seemed to bleed more and faster than patients with moderate to mild GCS.

### Other intracranial pathologies on baseline CT scans

TBI patients often present with intracranial pathologies in addition to intracranial bleeding. Compared to patients with mild to moderate GCS, the prevalence of sulcal effacement was greater in those with severe GCS (44% vs 59%; *n* = 190/433 vs *n* = 417/702), as was ventricular effacement (30% vs 47%; *n* = 128/433 vs 328/702), and midline shift (39% vs 48%; *n* = 169/433 vs. 337/702). Patients with a severe GCS and midline shift had a median shift of 7.4 mm (IQR 4.1–14.1 mm) whilst those with moderate to mild GCS had a median shift of 4.3 mm (IQR 2.8–7.1 mm).

### Intracranial bleeding on follow-up CT scans

Seventy-one percent (*n* = 812) of patients with a baseline CT scan had a second or third clinically indicated CT scan. Over a third of these patients (*n* = 318) had a bleed on a subsequent scan that was not seen on the first scan. Patients who had their first CT scan soon after injury were more likely to have a new bleed on a subsequent scan. The prevalence of new bleeds among those scanned ≤ 1.5 h,  > 1.5–3 h,  > 3–8 h after injury was 46%, 38%, 31%, respectively. For every 1 h increase from injury to the baseline scan, the risk of new bleeding on a further scan decreased by 12% (RR = 0·88 [95% CI 0.80–0.96], *p* = 0.0047) (adjusted for baseline GCS score, pupil reaction, and time from injury to follow-up scan). The sooner the first scan was done after injury, the greater the opportunity for a new bleed to manifest on a further scan.

### Baseline intracranial bleeding, raised intracranial pressure, un-reactive pupils, and head injury death

**Fig. 4:**
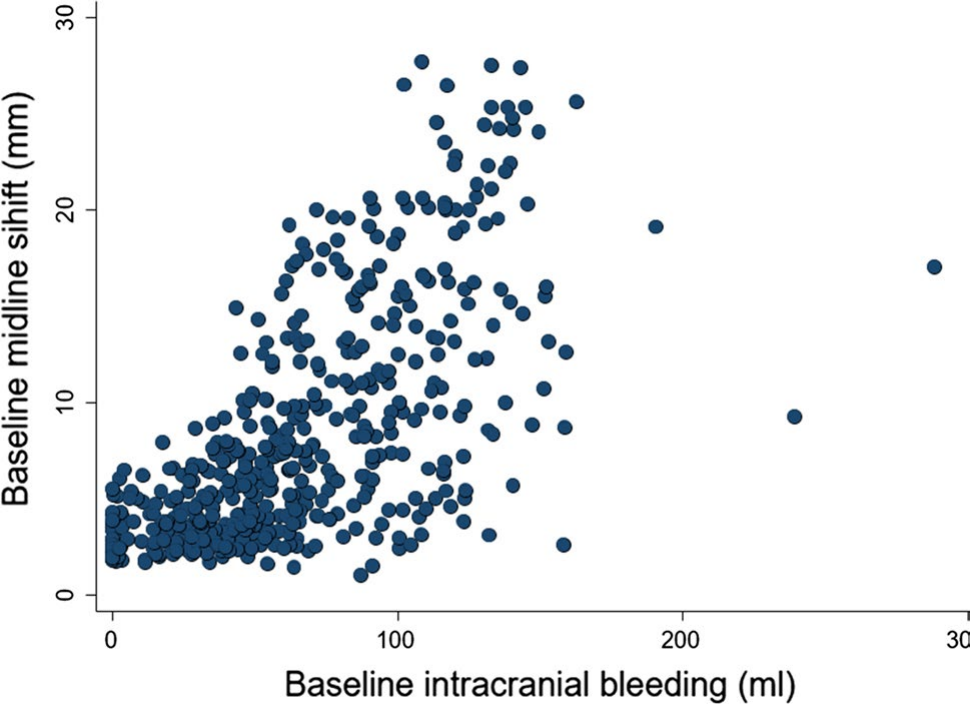
Association between baseline intracranial bleeding (ml) and baseline midline shift (mm)

**Fig. 5:**
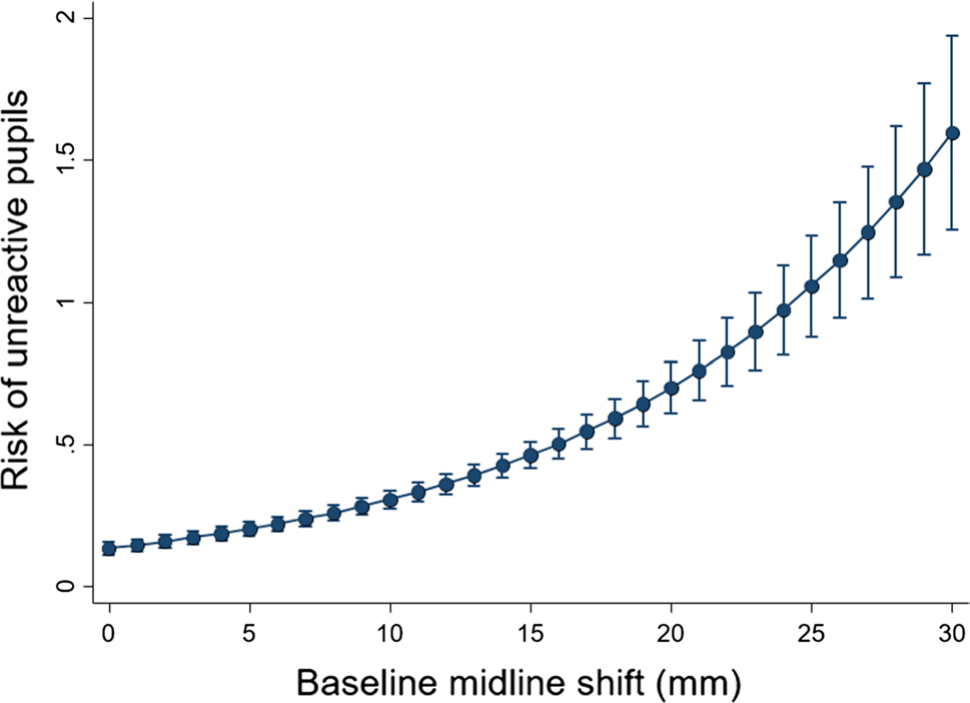
Association between midline shift and risk of un-reactive (compared to reactive) pupils at baseline

An increase in the volume of intracranial bleeding (ml) was associated with an increase in the amount (mm) of midline shift (beta coefficient 0.10 [95% CI 0.09–0.10], *p* < 0.0001) (see Fig. 4). An increase in midline shift (mm) was associated with an increase in the risk of having one or more un-reactive pupils (RR 1.08 [95% CI 1.07–1.10], *p* < 0.0001) (see Fig. 5). Of those with baseline scans available for rating, 247 patients subsequently died from head injury. The median time-adjusted volume of intracranial bleeding among patients who died from head injury is 37 ml/h (IQR 18–58 ml/h) and in those who did not die of head injury is 11 ml/h (IQR 0.3–28 ml/h). Patients who died of head injury within 24 h of injury had a higher median time-adjusted bleeding volume of 51 ml/h (IQR 28–73 ml/h), than those who died within 48–72 h of injury with 39 ml/h (IQR 19–56 ml/h), and beyond 72 h of injury with 28 ml/h (IQR 14–52 ml/h).

## Discussion

**Fig. 6:**
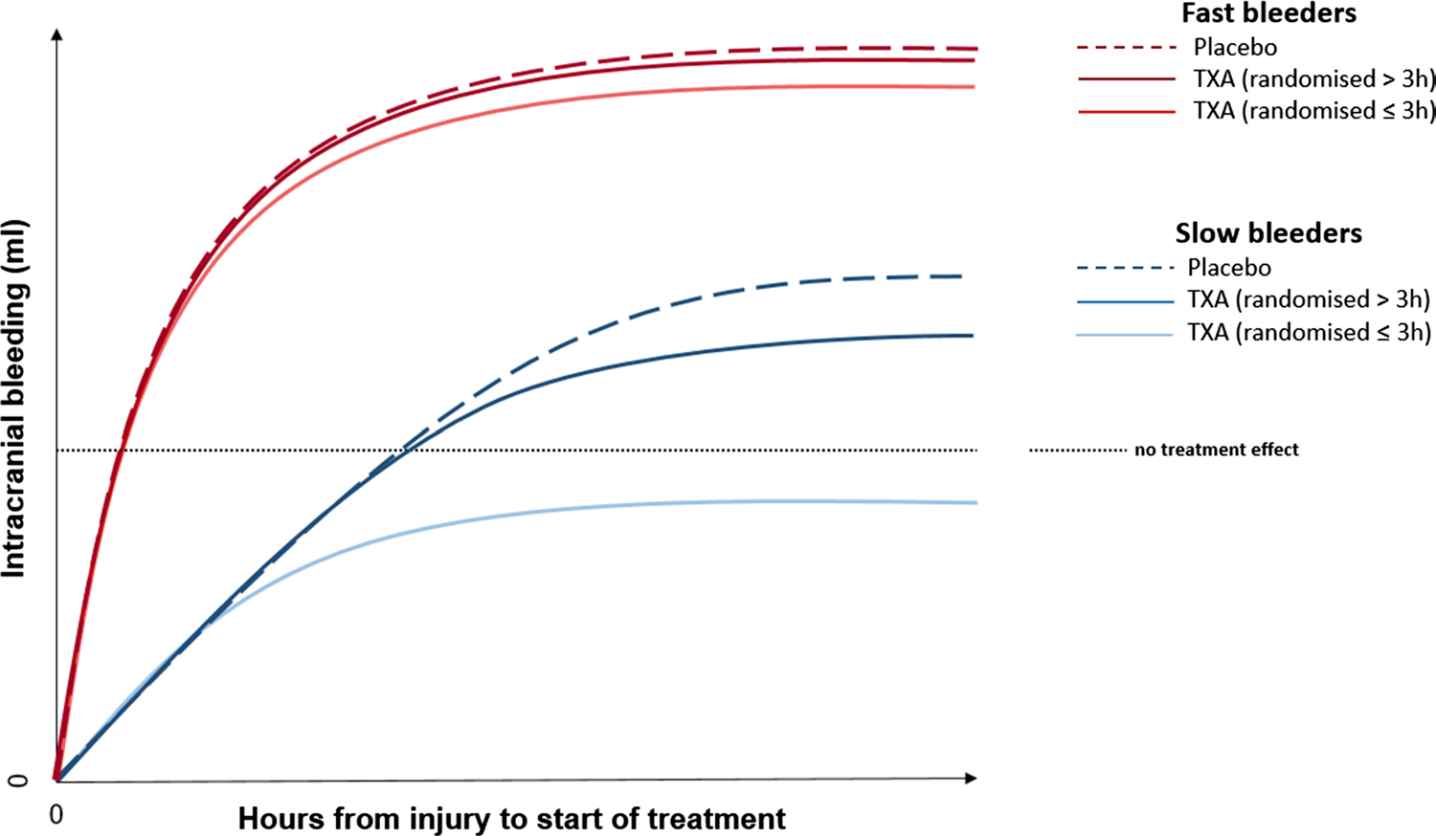
Hypothesis: association between bleeding rate and treatment effect

The CRASH-3 trial results suggest that the effect of tranexamic acid on head injury death depends on the time interval between injury and the start of treatment and on the severity of TBI [1]. Early treatment of patients with a mild to moderate GCS reduces head injury death, but there is no evidence for benefit in patients with a severe GCS, regardless of time to treatment. The CT scan data are consistent with the hypothesis that tranexamic acid reduces head injury deaths by reducing intracranial bleeding. Patients with a mild to moderate GCS may be more likely to benefit from tranexamic acid because they have less intracranial bleeding at baseline. However, because bleeding occurs soon after injury, treatment delay reduces the benefit. On the other hand, patients with a severe GCS have less to gain from treatment because they already have extensive intracranial bleeding at baseline and/or other intracranial pathologies that are not affected by tranexamic acid. Our explanatory hypothesis is illustrated in Fig. 6. Please note that this is not a figure of the treatment effect seen in this study.

The CRASH-3 investigators anticipated in their statistical analysis plan [6] that TBI patients with GCS 3 or bilateral un-reactive pupils at baseline would have little potential to benefit from tranexamic acid and their inclusion in the analysis would dilute any treatment effect towards the null. They, therefore, pre-specified a sensitivity analysis that excluded these patients. Our CT data supports this decision, showing that these patients have extensive intracranial bleeding, and other intracranial pathologies, prior to treatment. However, whilst patients with bilateral un-reactive pupils were excluded, those with unilateral un-reactive pupils were not, despite having high volumes of intracranial bleeding at baseline. Patients with unilateral un-reactive pupils might also have brain herniation and their inclusion might have diluted the treatment effect. Indeed, when patients with GCS 3 and any un-reactive pupils at baseline are excluded, the effect of tranexamic acid on head injury death is noticeably larger [1].

### Research in context

#### Evidence before this study

Although the risk of death and disability due to TBI may be reduced by preventing intracranial haemorrhage expansion [7–9], there is limited evidence on bleeding expansion, particularly according to bleed type. One study of 142 TBI patients with a median GCS of eight suggested that intracranial haemorrhage expansion varies according to haemorrhage type [10]. Repeat CT scans done within 24 h of injury suggested that IPH appeared to expand in 51% of patients, EDH in 22%, SAH in 17% and SDH in 11% of patients. But this study considered any expansion between first and second CT scans as evidence for expansion and did not measure the amount of expansion. The different eligibility criteria and definitions for expansion between studies make accurate estimation of expansion rates difficult. The decision for neurosurgical haemorrhage evacuation between first and second scans also complicates assessment of expansion rates. Furthermore, intracranial haemorrhage in its hyper-acute phase (before clotting) may not manifest on CT as its appearance is based on blood clot density changes over time [11–13]. Therefore, intracranial haemorrhage may have occurred by the point of the first CT scan, but not be visible. Studies that suggest that the prevalence of new bleeding on a second CT scan is greater when the first CT scan is done sooner after injury [7] may indicate that bleeding in its hyper-acute phase is not visible on a CT scan done very soon after injury [11–13], or that bleeding happens early. The absence of data on time from injury to scanning in many studies and the different times to scanning in studies that report these data limits understanding of the period over which expansion occurs or manifests on imaging.

The baseline CT data from the CRASH-3 IBMS improves understanding of the neuropathological presentation of TBI patients. The existing knowledge on intracranial haemorrhage, and other features of TBI, is based on smaller studies with different and restrictive inclusion criteria. The larger sample and less restrictive inclusion criteria of the CRASH-3 IBMS allowed this study to explore the natural occurrence of intracranial pathologies between injury and hospital admission. Since this is one of the largest descriptive studies conducted in TBI, the results should be useful for emergency physicians, neurosurgeons, and other clinical specialties. Furthermore, this information can inform the design and interpretation of clinical trials including patients across TBI severity.

#### Added value of this study

The findings from the current study may help explain the results of the largest randomised trial in TBI to date; the recently published CRASH-3 trial. If at baseline TBI patients present with intracranial bleeding and a number of other neuropathological changes that TXA cannot plausibly affect, their potential to benefit from TXA may reduce. Although clinical signs such as GCS score and pupil reaction were assessed at baseline, the CRASH-3 trial procedure did not involve examining the intracranial pathologies that may lead to these clinical signs. In severely injured patients, the immediate neurologic damage from the trauma may have been too severe to be alterable and TXA may have little potential to reduce intracranial bleeding progression and the risk of head injury death. In this study, we considered the occurrence of secondary neuropathological changes that occur after the primary TBI and before randomisation into the CRASH-3 trial. Knowledge of these changes can inform understanding of the potential for TXA to improve patient outcome and may help explain any variations in treatment effect by baseline injury severity in the CRASH-3 trial.

#### Implications of all the available evidence

The CRASH-3 trial treatment was given after arrival at hospital. Less than 20% of patients were treated within an hour of injury. Although there was no apparent benefit in patients with a low GCS on hospital arrival, if our explanatory hypothesis is correct, some of these patients might have benefited had they been treated in the pre-hospital setting. In many high-income countries, TXA is routinely administered by paramedics at the scene of the injury to treat acute severe bleeding. In low- and middle-income settings, this is not always possible due to resource constraints and a lack of health workers who can administer intravenous drugs in the pre-hospital setting. Alternatives to intravenous administration of TXA such as intramuscular injection would be easier, require less training, and may reduce time to treatment. However, patients with more severe injuries in settings with insufficient in-hospital resources may die despite an early reduction in intracranial bleeding. Evidence suggests that patients with severe TBI in low- and middle-income settings may be more likely to die compared to those in high-income settings. More rapid administration of TXA in settings with adequate medical care for patients with major trauma could increase the proportion of TBI patients who have the potential to benefit.

## Abbreviations

CI: Confidence interval
CRASH-3: Clinical Randomisation of an Antifibrinolytic in Significant Head Injury
CT: Computed tomography
EDH: Epidural haemorrhage
GCS: Glasgow coma score
IBMS: Intracranial bleeding mechanistic study
IPH: Intra-parenchymal haemorrhage
IQR: Interquartile range
IVH: Intra-ventricular haemorrhage
RR: Relative risk
SDH: Subdural haemorrhage
TBI: Traumatic brain injury
